# Classification of Riboswitch Families Using Block Location-Based Feature Extraction (BLBFE) Method

**DOI:** 10.15171/apb.2020.012

**Published:** 2019-12-11

**Authors:** Faegheh Golabi, Mousa Shamsi, Mohammad Hosein Sedaaghi, Abolfazl Barzegar, Mohammad Saeid Hejazi

**Affiliations:** ^1^Genomic Signal Processing Laboratory, Faculty of Biomedical Engineering, Sahand University of Technology, Tabriz, Iran.; ^2^School of Advanced Medical Sciences, Tabriz University of Medical Sciences, Tabriz, Iran.; ^3^Faculty of Electrical Engineering, Sahand University of Technology, Tabriz, Iran.; ^4^Research Institute for Fundamental Sciences (RIFS), University of Tabriz, Tabriz, Iran.; ^5^Molecular Medicine Research Center, Biomedicine Institute, Tabriz University of Medical Sciences, Tabriz, Iran.; ^6^Faculty of Pharmacy, Tabriz University of Medical Sciences, Tabriz, Iran.

**Keywords:** Riboswitch, Non-coding RNA, Sequential blocks, Block location-based feature extraction, BLBFE, Classification, Performance measures

## Abstract

***Purpose:*** Riboswitches are special non-coding sequences usually located in mRNAs’ un-translated regions and regulate gene expression and consequently cellular function. Furthermore, their interaction with antibiotics has been recently implicated. This raises more interest in development of bioinformatics tools for riboswitch studies. Herein, we describe the development and employment of novel block location-based feature extraction (BLBFE) method for classification of riboswitches.

***Methods:*** We have already developed and reported a sequential block finding (SBF) algorithm which, without operating alignment methods, identifies family specific sequential blocks for riboswitch families. Herein, we employed this algorithm for 7 riboswitch families including lysine, cobalamin, glycine, SAM-alpha, SAM-IV, cyclic-di-GMP-I and SAH. Then the study was extended toward implementation of BLBFE method for feature extraction. The outcome features were applied in various classifiers including linear discriminant analysis (LDA), probabilistic neural network (PNN), decision tree and k-nearest neighbors (KNN) classifiers for classification of the riboswitch families. The performance of the classifiers was investigated according to performance measures such as correct classification rate (CCR), accuracy, sensitivity, specificity and f-score.

***Results:*** As a result, average CCR for classification of riboswitches was 87.87%. Furthermore, application of BLBFE method in 4 classifiers displayed average accuracies of 93.98% to 96.1%, average sensitivities of 76.76% to 83.61%, average specificities of 96.53% to 97.69% and average f-scores of 74.9% to 81.91%.

***Conclusion:*** Our results approved that the proposed method of feature extraction; i.e. BLBFE method; can be successfully used for classification and discrimination of the riboswitch families with high CCR, accuracy, sensitivity, specificity and f-score values.

## Introduction

Regulation of cellular functions are achieved by effective collaboration of varying types of bio-molecules such as DNAs, RNAs and proteins. Riboswitches^1-4^ as an example of regulatory RNAs, are a part of mRNA molecules and regulate the expression of corresponding genes by directly binding to the target metabolites and undergoing consequent structural changes.^5-7^ For instance, the riboswitch structural conformation alteration blocks the ribosome binding site and inhibits protein synthesis by the ribosome. Riboswitches are usually located in mRNAs’ 5’ un-translated regions.^3^ Riboswitches with similar sequence and secondary and tertiary structures perform similar tasks.^8,9^ Therefore, riboswitches are categorized to families according to their function, sequence conservation and structural similarities.^10,11^

Studies showed that riboswitches interact with antibiotics and regulate the expression of the corresponding gene. The interaction of antibiotics with riboswitches could be attributed at least partly to the action mechanism of the antibacterial agents.^12^ Sudarsan and colleagues^13^ showed the interaction of pyrithiamine with thiamine pyrophosphate riboswitch. Interaction of lysine riboswitch with antibiotics was reported by Blount and co-workers^14^ and interaction of roseoflavin antibiotic with FMN riboswitch was also confirmed.^15-17^ Our *in-silico* studies indicated that aminoglycosides including kanamycin interact with various riboswitches and their binding energies are comparable or sometimes higher than those of their native target molecules.^18,19^ Later, Baird and colleagues^20^ during their study, unexpectedly and interestingly noticed that kanamycin binds to cyclic diguanylate (cyclic-di-GMP) riboswitch and inhibits its binding with native ligand. Their *in-vitro* findings were in accordance with our *in-silico* results. Riboswitches could be considered as new targets for antibiotics and their interaction with antibiotics could explain new mechanisms for antibiotics’ functions and effects and consequently opens a new era for development of novel antibiotics. Established important role of riboswitches in the nature and development of novel therapeutic agents attracts increased attention for elucidation of riboswitches’ characteristics and development of new tools for riboswitch detection is accordingly in demand.

Classification of riboswitches into their related families gives insight to their functionality and structural aspects. One of the common principles for classification of riboswitches relies on homology search.^21^ Based on this principle, various statistical methods have been developed such as hidden Markov models based methods^22-25^ and CM or covariance model.^26^ Singh and Singh used mononucleotide and dinucleotide conservation based features to classify the riboswitches.^27^ Pse-in-One web server also generates various modes of pseudo components of RNA sequences which can be used as feature vectors for classification of riboswitches.^28,29^

A non-alignment sequential block finding algorithm (SBF) was designed for identification of family specific RNA sequential blocks in different riboswitch families.^30^ In the present study, we applied the SBF to 7 families of riboswitches and extracted the family specific sequential blocks. Then, we developed BLBFE method as a novel feature extraction method based on the locations of the detected blocks. The extracted features were utilized for classification of the riboswitches. For this, linear discriminant analysis (LDA),^31^ probabilistic neural network (PNN),^32^ decision tree^33^ and k-nearest neighbors (KNN)^34^ classifiers accompanied by V-fold cross-validation^35^ were applied for classification of sequences into their related classes (families) based on the features extracted by block location-based feature extraction (BLBFE) method. Then, the performance of each classifier was presented by a confusion matrix. In the next step, performance measures such as accuracy, sensitivity, specificity and f-score were calculated for each classifier to study the performance validity of the developed feature extraction method.

## Materials and Methods

### Datasets

Table 1 shows seven families of riboswitches, whose seed data were used for block detection and classification in this study. The riboswitch families include lysine,^36,37^ cobalamin,^7,38-40^ glycine,^41-43^ SAM-alpha,^44,45^ SAM-IV,^46,47^ cyclic-di-GMP-I^46,48,49^ and SAH^50,51^ families, containing 47, 430, 44, 40, 40, 155 and 52 seed members in each family, respectively. Datasets along with their sequential and secondary structure characteristics were downloaded from Rfam 13.0 database in un-gapped FASTA format.^52,53^ Table 1 also represents calculated mean lengths and variance of lengths of the members for the studied families.

**Table 1:** Seven riboswitch families obtained from Rfam 13.0 database

**Riboswitch family name**	**Rfamaccession number**	**Number of seed data**	**Average length of members (nucleotides)**	**Variance of the length of members**
Lysine	RF00168	47	183	11.06
Cobalamin	RF00174	430	203	15.54
Glycine	RF00504	44	101	15.99
SAM-alpha	RF00521	40	79	1.18
SAM-IV	RF00634	40	116	4.13
Cyclic-di-GMP-I	RF01051	155	87	6
SAH	RF01057	52	85	15.4

### Application of the block finding algorithm

We have previously designed a block finder program for detection of frequent RNA blocks in riboswitch families.^30^ In this method, an algorithm was used to identify the frequently appearing specific sequential blocks in riboswitch families. These blocks are characteristic motifs of a certain riboswitch family which are present in a very high percentage of the riboswitch family members complying the sequence conservation of riboswitch families. Also in a high percentage of family members, location of the motifs on the sequences should be the same or in a close defined neighborhood. In this path, the algorithm first recognizes all potential blocks, then checks each block’s location on every member of the family and eliminates the excess blocks accordingly. Finally, for each riboswitch family a set of specific sequential blocks is determined.

### Feature extraction

We employed the locations of family-specific blocks on riboswitch sequences as features for classification of the riboswitches. To extract the features, first, sequential conserved blocks for the seven riboswitch families were detected using our previously reported SBF method. The detected blocks were then employed to produce observations related to each riboswitch family member. For this, the start point of each block on the sequence was considered as the block’s location in the sequence. Then, the locations of the blocks in the sequences were used as features. For the blocks which were not present in the sequence, the location was set to zero. For example, to produce an observation based on the following 10 blocks [GGUUC, CCC, AAAAACUA, GUGC, UAUA, UCUACC, GGGC, GGAUG, GGG, CUGAGA] for a sample riboswitch sequence such as:

“CCGCAUUCUCAGGGCAGCGU GAAAUUCCCUACU GGCGGUCAAGCG CGCGAGCGUU UGUUAUAAGGCAAAU CAGCAGAUUUGGUGAAAU UCCAAAGCCAA CAGUUACA GUCUGGA UGAAAG AGAGUAAAC”

The location of each block on the sequence is determined. Since block “GGUUC” (the first block) is not present in the sequence, its location is set to zero. The block “CCC” (the second block) starts from the 27^th^ nucleotide of the sequence, so its location is set to 27. “AAAAACUA” and “GUGC” blocks are also not present in the sequence and their locations are considered as zero. The “UAUA” block is seen at the 59^th^ nucleotide. Similarly, “UCUACC”, “GGGC”, “GGAUG”, “GGG” and “CUGAGA” blocks are located at 0^th^, 12^th^, 113^th^, 12^th^ and 0^th^ nucleotides, respectively. By finding the location of the all blocks, the observation associated with the above mentioned sequence is [0, 27, 0, 0, 59, 0, 12, 113, 12, 0] which is a 1 by 10 array. Accordingly, for each riboswitch sequence in each family, an observation is generated based on detected sequential blocks. In other word, each sequence is demonstrated by an observation. Therefore, the overall number of observations equals to the total number of the 7 riboswitch families’ members and the length of each array is equal to the number of total blocks for 7 families. The observations are then utilized for classification of the sequences into their associated families.

### Cross-validation

To validate the generalization of the classifiers, V-fold cross-validation (VFCV) was used.^35^ VFCV, due to its mild computational cost, is the most popular CV procedure. For a dataset with N members, VFCV partitions the data randomly into V subsets with approximately equal cardinality of N/V. Each subset successively plays the role of test data while the rest of the data is used to train the classifier. The overall correct classification rate (CCR) is average of the CCRs of the V stages. Here, V=10 was used for cross-validation because of the good error estimation in addition to suitably low computational cost.^31,54-56^

### The classifiers

Four classifiers were employed to study the performance of the proposed feature extraction method.

*Linear discriminant analysis (LDA) classifier:* This method finds a linear combination of features to characterize or discriminate two or more classes and uses the resulting combination as a linear classifier. The LDA method is a generalization of Fisher’s linear discriminant.^31^

*Probabilistic neural network (PNN) classifier:* The PNN algorithm estimates the class probability of an input data using the probability distribution function of each class. Then Bayes’ rule is employed to assign the input data to the class with highest posterior probability.^32^

*Decision tree classifier:* Decision tree method creates a predictive tree-like model using a series of carefully created questions. Based on the tree as the model, it goes from observations about an input data, represented by the branches of the tree, to decisions about the input data’s class label, represented by the leaves.^33^

*K-nearest neighbors (KNN) classifier:* In KNN classification, an input data is classified to the class most common among its K nearest neighbors. K is a positive integer number, usually small.^34^ In this study, the optimum K was equaled to 4.

### Evaluation of classifiers’ performance

Four performance measures of accuracy, sensitivity, specificity and f-score are calculated according to the confusion matrices using the equations (1) to (4)^57-59^:

(1) $$\text{Accuracy}=\frac{TP+TN}{TP+FP+TN+FN}$$

(2) $$\text{Sensitivity}=\frac{TP}{TP+FN}$$

(3) $$\text{Specificity}=\frac{TN}{FP+TN}$$

(4) $$\text{F-score}=\frac{2TP}{2TP+FP+FN}$$

TP denotes the true positive rate; i.e. the members of each class which are correctly classified to the right class. FP is the false positive rate; i.e. the sequences which are falsely annotated to another class. Also, TN and FN are the true negative and the false negative rates, respectively.

## Results and Discussion

### Detection of family specific blocks

Frequently appearing RNA sequential blocks for seven riboswitch families were detected using SBF method.^30^ Results of the block finder algorithm for 7 families are presented in Table 2.

**Table 2:** Results of the application of the sequential block finding (SBF) algorithm for 7 families of riboswitches

**Riboswitch family name**	**Blocks**	**Approximate Location on the sequences**
Lysine	AGAGGUGC	10
	AGUAA	28
Cobalamin	CGGUG	18
	GCA	77
	AGC	92
	AGA	175
	GACC	180
Glycine	GGAGA	13
	CCGA	35
SAM-alpha	GUGGU	11
	AUUUG	17
GCCACGU	37
SAM-IV	UCA	3
	GAG	7
	CAG	13
	GCUGG	32
	CGGCAACC	38
Cyclic-di-GMP-I	GAAA	23
	CGCAAAGC	35
SAH	GAGGAGCG	7
	UGC	16
	AGGCUCGG	36

As can be seen, our algorithm detected 2 blocks for the lysine family including ‘AGAGGUGC’ and ‘AGUAA’ blocks at locations 10 and 28, respectively. For the cobalamin family, 5 blocks including ‘CGGUG’, ‘GCA’, ‘AGC’, ‘AGA’ and ‘GACC’ were recognized which are located at locations 18, 77, 92, 175 and 180, respectively. Also, 2 blocks were detected for the glycine family including ‘GGAGA’ and ‘CCGA’ recognized at locations 13 and 35, respectively. For the SAM-alpha riboswitch family, 3 blocks including ‘GUGGU’, ‘AUUUG’ and ‘GCCACGU’ were recognized at locations 11, 17 and 37, respectively. Five specific blocks were detected for SAM-IV family including ‘UCA’, ‘GAG’, ‘CAG’, ‘GCUGG’ and ‘CGGCAACC’ blocks located at 3, 7, 13, 32 and 38 locations, respectively. For cyclic-di-GMP-I family, 2 blocks of ‘GAAA’ located at 23 and ‘CGCAAAGC’ located at 35 nucleotides were identified. And finally 3 blocks were detected for SAH family including ‘GAGGAGCG’, ‘UGC’ and ‘AGGCUCGG’ located at locations 7, 16 and 36, respectively. Therefore, 22 sequential blocks were identified for 7 studied riboswitch families, in total.

### Model validation

**Figure 1:**
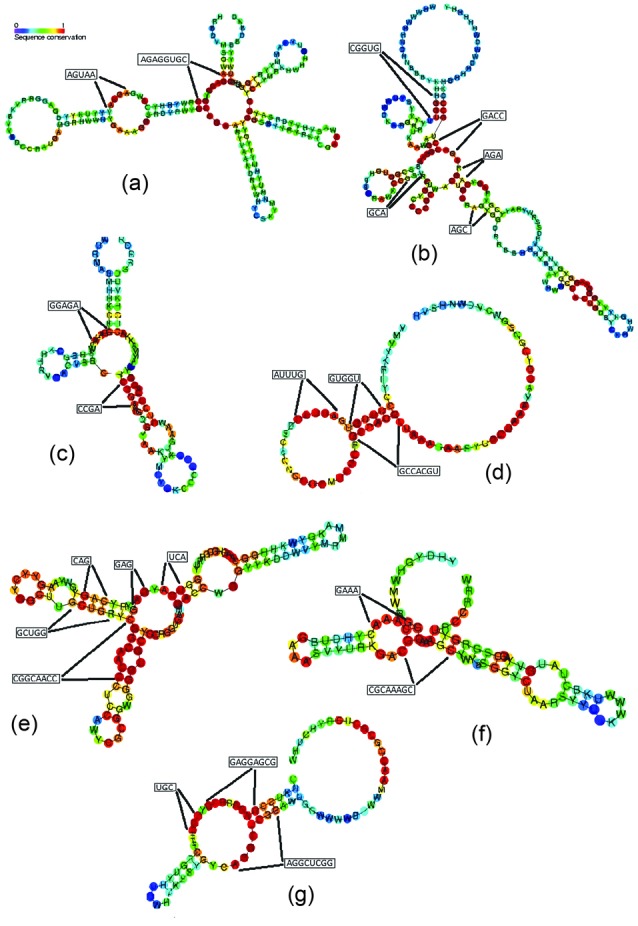
Accordance of the detected family specific blocks to the conserved regions of secondary structures of studied riboswitch families (based on the Rfam database). The diagrams are related to secondary structures of: (a) lysine, (b) cobalamin, (c) glycine, (d) SAM-alpha, (e) SAM-IV, (f) cyclic-di-GMP-I and (g) SAH riboswitches.

Our results for 7 riboswitch families were compared to the conserved regions observed in the alignment results from Rfam database (Figure 1). As seen, most of the detected blocks fall into the highly conserved regions (shown in red) in the studied families. For example, two 8 and 5-mer blocks, ‘AGAGGUGC’ and ‘AGUAA’, were detected for lysine family. As shown in Figure 1a, these blocks are located exactly in the highly conserved areas of the lysine riboswitch structure. Also, Figures 1b-1g demonstrate the accordance of the detected blocks for cobalamin, glycine, SAM-alpha, SAM-IV, cyclic-di-GMP-I and SAH riboswitches with the consensus segments of the families, respectively.

### Classification results

Using sequential based block finding algorithm, SBF, 22 RNA sequential blocks [AGAGGUGC, AGUAA, CGGUG, GCA, AGC, AGA, GACC, GGAGA, CCGA, GUGGU, AUUUG, GCCACGU, UCA, GAG, CAG, GCUGG, CGGCAACC, GAAA, CGCAAAGC, GAGGAGCG, UGC, AGGCUCGG] were detected and determined as family specific blocks for 7 families. Having detected the specific blocks, observations were created using BLBFE method for classification of the riboswitches. Locations of these blocks on the family members are considered as features. The resulted 1 by 22 arrays are observations, each representing one of the riboswitches for designed classifier. As there are 808 members in total in 7 studied riboswitch families, 808 arrays of 1 by 22 as observations were produced. Of 808 created observations, 47, 430, 44, 40, 40, 155 and 52 ones belong to lysine, Cobalamin, Glycine, SAM-alpha, SAM-IV, Cyclic-di-GMP-I and SAH families, respectively. For each set of observations, LDA, PNN, decision tree and KNN classifiers accompanied by 10-fold cross-validation were applied. Then, correct and incorrect classified samples for each set were counted.

**Figure 2:**
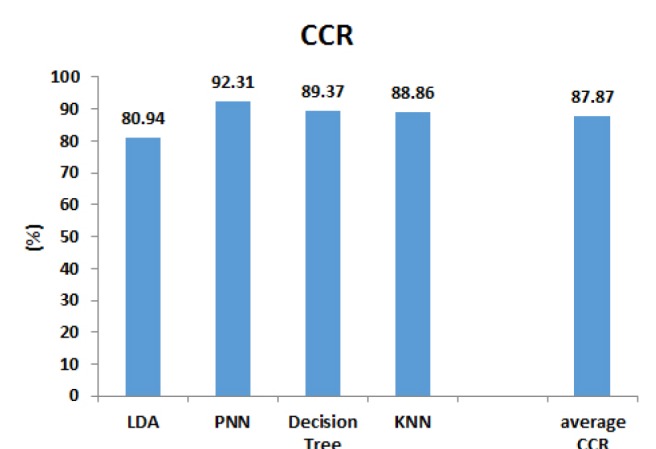
Correct classification rates for 4 classifiers using block location-based feature extraction method (BLBFE).

Figure 2 shows the correct classification rates (CCRs) of the studied classifiers. With the BLBFE method, PNN with 92.31% had the highest CCR while the other classifiers showed CCRs of 89.37% for decision tree classifier, 88.86% for KNN classifier and finally 80.94% for LDA classifier. Overall, the average CCR of four classifiers when using locations of specific sequential blocks as features was 87.87%.

### Evaluation results

**Figure 3:**
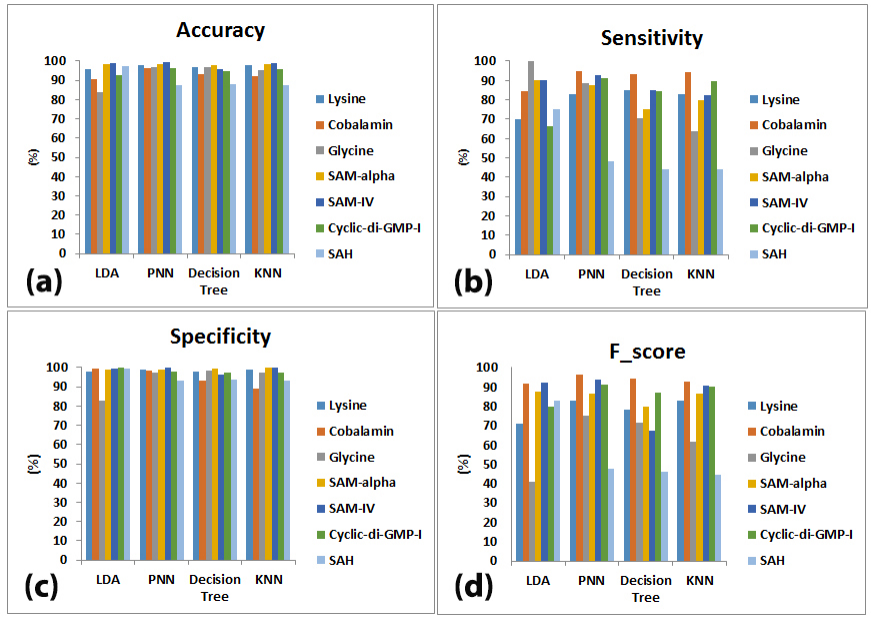
The performance measures for classification of 7 riboswitch families by LDA, PNN, decision tree and KNN classifiers using block location-based feature extraction method (BLBFE), (a) accuracy, (b) sensitivity, (c) specificity and (d) f-score.

Table 3 represents the multiclass confusion matrix for LDA classifier with the BLBFE. Also, the multiclass confusion matrices for PNN, decision tree and KNN classifiers with the same method of feature extraction are represented in Tables 4 to 6, respectively. Based on the confusion matrices, accuracy, sensitivity, specificity and f-score measures for the BLBFE method were calculated and illustrated in Figure 3.

**Table 3:** Multiclass confusion matrix for the LDA classifier, based on the features extracted by the block location-based feature extraction (BLBFE) method

**Predicted/True Riboswitch Families**	**Lysine**	**Cobalamin**	**Glycine**	**SAM-alpha**	**SAM-IV**	**Cyclic-di-GMP-I**	**SAH**
Lysine	33	1	10	3	0	0	0
Cobalamin	13	363	46	3	1	1	3
Glycine	0	0	44	0	0	0	0
SAM-alpha	0	0	4	36	0	0	0
SAM-IV	0	0	4	0	36	0	0
Cyclic-di-GMP-I	0	0	52	0	0	103	0
SAH	0	0	12	0	1	0	39
TP	33	363	44	36	36	103	39
FP	13	1	128	6	2	1	3
TN	621	291	610	618	618	551	615
FN	14	67	0	4	4	52	13

**Table 4:** Multiclass confusion matrix for the PNN classifier, based on the features extracted by the block location-based feature extraction (BLBFE) method

**Predicted/True Riboswitch Families**	**Lysine**	**Cobalamin**	**Glycine**	**SAM-alpha**	**SAM-IV**	**Cyclic-di-GMP-I**	**SAH**
Lysine	39	1	4	0	0	1	2
Cobalamin	6	407	7	3	1	5	1
Glycine	1	1	39	0	0	3	0
SAM-alpha	0	1	1	35	1	2	0
SAM-IV	0	1	0	1	37	1	0
Cyclic-di-GMP-I	1	2	7	2	0	141	2
SAH	0	0	2	0	0	2	48
TP	39	407	39	35	37	141	48
FP	8	6	21	6	2	14	53
TN	707	339	707	711	709	605	698
FN	8	23	5	5	3	14	52

**Table 5:** Multiclass confusion matrix for the decision tree classifier, based on the features extracted by the block location-based feature extraction (BLBFE) method

**Predicted/True Riboswitch Families**	**Lysine**	**Cobalamin**	**Glycine**	**SAM-alpha**	**SAM-IV**	**Cyclic-di-GMP-I**	**SAH**
Lysine	40	4	1	0	0	1	1
Cobalamin	11	402	8	1	7	1	0
Glycine	1	6	31	1	0	5	0
SAM-alpha	0	4	0	30	4	2	0
SAM-IV	1	0	1	1	34	3	0
Cyclic-di-GMP-I	1	7	2	1	11	131	2
SAH	1	1	0	1	5	3	41
TP	40	402	31	30	34	131	41
FP	15	22	12	5	27	15	44
TN	669	307	678	679	675	578	668
FN	7	28	13	10	6	24	52

**Table 6:** Multiclass confusion matrix for the KNN classifier, based on the features extracted by the block location-based feature extraction (BLBFE) method

**Predicted/True Riboswitch Families**	**Lysine**	**Cobalamin**	**Glycine**	**SAM-alpha**	**SAM-IV**	**Cyclic-di-GMP-I**	**SAH**
Lysine	39	3	2	0	0	2	1
Cobalamin	4	406	9	1	0	7	3
Glycine	0	14	28	0	0	2	0
SAM-alpha	0	5	1	32	0	1	1
SAM-IV	1	5	0	0	33	0	1
Cyclic-di-GMP-I	2	9	2	1	0	139	2
SAH	1	2	5	0	0	3	41
TP	39	406	28	32	33	139	41
FP	8	38	19	2	0	15	49
TN	679	312	690	686	685	579	677
FN	8	24	16	8	7	16	52

As seen in Figure 3a, classification accuracy measures for LDA classifier is ranged between 99.09% for SAM-IV and 83.63% for glycine families. For PNN classifier, SAM-IV family again displays the highest accuracy of 99.33% while the lowest accuracy is 87.66% for SAH family. Decision tree classifier has maximum accuracy of 97.93% for SAM-alpha family and minimum accuracy of 88.07% for SAH family. At last, the highest accuracy for KNN classifier is 99.03% which belongs to SAM-IV family, and the lowest accuracy is 87.67% for SAH family.

Figure 3b shows individual sensitivities of 4 classifications. The LDA classifier resulted in sensitivity of 100% for glycine family while the lowest sensitivity is 66.45% for cyclic-di-GMP-I family. For PNN classifier, sensitivities are ranged between 94.65% for cobalamin and 48% for SAH families. The highest sensitivity for decision tree classifier is 93.49% for cobalamin family and the lowest is 44.09% for SAH family. Finally, KNN classifier results in sensitivities from 94.42% for cobalamin to 44.09% for SAH families.

The specificities of 4 classifiers are demonstrated in Figure 3c. As demonstrated, the highest specificity for LDA classifier is 99.82% belonging to cyclic-di-GMP-I family and glycine family has the lowest specificity of 82.66%. For PNN classifier, specificities range from 99.72% for SAM-IV to 92.94% for SAH families. SAM-alpha has the highest of 99.27% with decision tree classifier while cobalamin has the lowest specificity of 93.31%. For KNN classifier, the highest specificity, 100% belongs to SAM-IV family and the lowest is 89.14% belonging to cobalamin family.

Finally, Figure 3d presents the f-scores of 4 classifiers. For LDA classifier, f-scores range from 92.31% for SAM-IV to 40.74% for glycine families. The highest f-score with PNN classifier is 96.56% belonging to cobalamin family while the lowest f-score is 47.76% for SAH family. Application of decision tree classifier results in f-scores from 94.15% for cobalamin family to 46.07% for SAH. The KNN classifier also gives maximum f-score of 92.91% for cobalamin family in addition to minimum f-score of 44.81% for SAH family.

**Figure 4:**
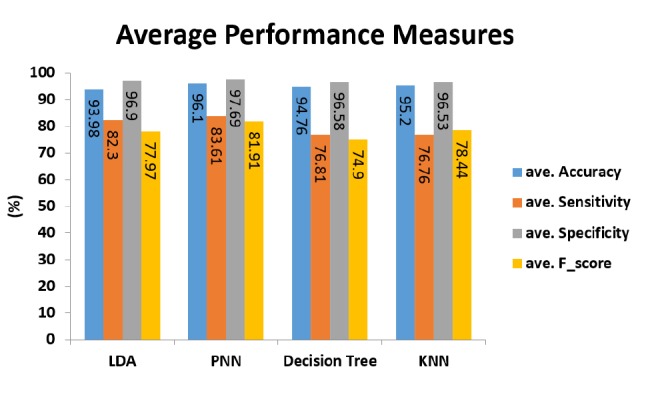
The average performance measures of 7 riboswitch families for the proposed method of BLBFE applied in 4 classifiers.

Figure 4 shows the average performance measures of 7 riboswitch families for the BLBFE method applied in 4 classifiers. As can be seen, PNN classifier has the best average accuracy, equal to 96.1%. This is while, other classifiers also represent good average accuracies of 95.2% for KNN classifier, 94.79% for decision tree classifier and 93.98% for LDA classifier. PNN classifier has the highest average sensitivity too. 83.61%, 82.3%, 76.81% and 76.76% are average sensitivities of PNN, LDA, decision tree and KNN classifiers, respectively.

The highest average specificity, 97.69%, belongs to the PNN classifier, followed by 96.9%, 96.58% and 96.53% for LDA, decision tree and KNN classifiers, respectively. Finally, PNN classifier again has the best average f-score, 81.91%. Other classifiers display average f-scores of 78.44% for KNN classifier, 77.97% for LDA classifier and 74.9% for decision tree classifier.

## Conclusion

The importance of riboswitches’ role in gene expression regulation and their interaction with antibiotics, have attracted more interest for development of new bioinformatics tools for recognition and characterization of riboswitches. Following development of SBF algorithm for detection of frequently appearing family specific sequential blocks in riboswitch families, in this paper we first elucidated the performance of the designed algorithm in detection of the family related blocks in lysine, cobalamin, glycine, SAM-alpha, SAM-IV, cyclic-di-GMP-I and SAH riboswitches. Results showed that the developed method detected most of the conserved motifs present in each family defined as family specific blocks. Then, the identified blocks on riboswitch sequences were used for classification of the members into their corresponding families. For this, we proposed a new feature extraction strategy called BLBFE, which employs the locations of the specified blocks on riboswitch sequences as features. Therefore, each riboswitch sequence is converted into a numerical array called an observation. In order to validate the performance of the proposed feature extraction method, 4 popular classifiers including LDA, PNN, decision tree and KNN were applied and their functions in classification of the riboswitches were evaluated and compared. Putting together the results, the BLBFE strategy led to suitable performance in classification of the riboswitches with average CCR of 87.87%. Having applied BLBFE, all the studied classifiers displayed closely suitable performances, where PNN classifier performed the best according to its higher accuracy, sensitivity, specificity and f-score. Considering the proposed BLBFE method’s performance, it is concluded that the developed methods of SBF and BLBFE are promising strategies for classification of the riboswitches. More reports from our group in development and application of the BLBFE method for other groups of RNAs, DNAs and genes are in progress.

## Ethical Issues

Not applicable.

## Conflict of Interest

Authors declare no conflict of interest in this study.
